# Non-erythrocyte spectrin network preferentially stabilizes flat membrane and enhances cell stiffness

**DOI:** 10.1039/d5ra08200e

**Published:** 2025-12-12

**Authors:** Takumi Komikawa, Takahiro Miyake, Mayu Morooka, Rikuto Kawakami, Shogo Saito, Kevin Critchley, Stephen D. Evans, Mina Okochi, Masayoshi Tanaka

**Affiliations:** a School of Materials and Chemical Technology, Institute of Science Tokyo 4259 Nagatsuda, Midori-ku Yokohama-city Kanagawa 226-8503 Japan tanaka.m@mct.isct.ac.jp +81-45-924-5567 +81-45-924-5567; b School of Materials and Chemical Technology, Institute of Science Tokyo 2-12-1 O-okayama Meguro-ku Tokyo 152-8552 Japan okochi.m.aa@m.titech.ac.jp +81-3-5734-2116 +81-3-5734-2116; c School of Physics and Astronomy, University of Leeds Leeds LS2 9JT UK

## Abstract

Spectrin αII and βII, the predominant non-erythroid isoforms, assemble into cytoskeletal networks that shape the plasma membrane. However, how these networks interact with membranes of different curvatures remains unclear. Using microfluidic deformation cytometry, we show that spectrin βII overexpression increases the apparent stiffness of MDA-MB-231 breast cancer cells. Fluorescence microscopy further demonstrates that spectrin is excluded from highly curved regions and enriched on flatter membranes *in vivo*, specifically those with an absolute curvature |*κ*| < 0.2 µm^−1^. Consistently, *in vitro* reconstitution with spherical supported lipid bilayers (SSLBs) shows that purified spectrin heterodimers preferentially bind low-curvature membranes, exhibiting ∼15-fold higher association with 1000 nm SSLBs (|*κ*| ≈ 0.5 µm^−1^) than with 30 nm SSLBs (|*κ*| ≈ 66.7 µm^−1^). This common curvature-dependent preference is promoted by spectrin oligomerization. Together, these results establish spectrin as a curvature-responsive cortical scaffold that selectively stabilizes flat membrane domains, thereby maintaining cellular stiffness.

## Abstract 2

The combination of microfluidic deformability cytometry and a spherical supported lipid bilayer assay reveals that spectrin is a key determinant of cell stiffness, and that its localization depends on membrane topology.

## Graphical abstract

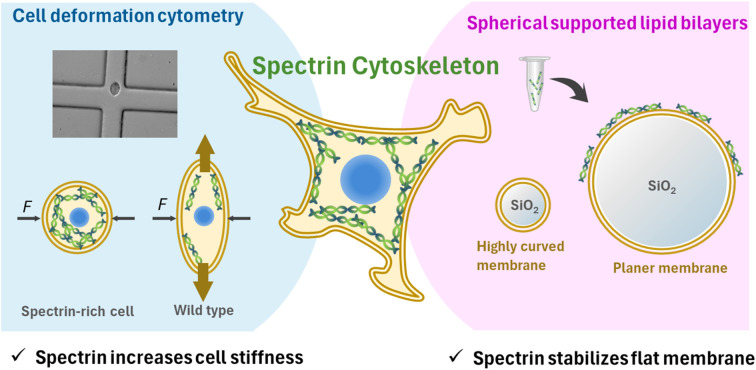

## Introduction

Eukaryotic cells regulate their plasma membrane morphology and mechanical properties, including stiffness and deformability, through a coordinated cytoskeletal system that includes actin and spectrin. Cellular stiffness is broadly reduced across cancer types, and this universal mechanobiological change has emerged as a promising diagnostic marker.^1–4^ During the transition from normal to motile cancer cells, cytoskeletal remodeling decreases the overall stiffness and enhances metastatic potential (*i.e.*, migration and invasiveness).^5^ The actin cortex, for example, plays a central role in maintaining the shape and stiffness of healthy cells.^6,7^ Spectrin, another major cytoskeletal protein expressed in most eukaryotic cells, also contributes to preserving cell morphology and polarity.^8^ Spectrin forms α-β heterodimers composed of repetitive spectrin domains, which further assemble into tetramers that link to actin filaments *via* the β-subunit's actin-binding domain. This organization gives rise to spectrin–actin networks that support the plasma membrane and regulate its architecture.^8^ This submembrane cytoskeletal scaffold not only supports the plasma membrane but may also constrain dynamic morphological processes. For example, the spectrin–actin network has been suggested to restrict the spatial positioning of clathrin-mediated endocytosis in proximal axons^9^ and to participate in lipid raft–dependent endocytosis of the close homolog of adhesion molecule L1 in neurons.^10^ In addition, redistribution of spectrin to the cytosol has been associated with enhanced membrane blebbing and vesiculation.^11^

The mechanical role of spectrin-based submembrane networks has been extensively studied in red blood cells and neurons^10,12,13^ but their functions and role in cellular mechanobiology in non-erythroid cells remain less defined. Spectrin and actomyosin together form complementary mesoscale cortical networks that stabilize the membrane cortex.^14^ Furthermore, the expression levels of spectrin αII (SPTAN1) and βII (SPTBN1), the most abundant isoforms in non-erythroid cells, change across different cancer types.^15,16^ In breast cancer cells (MCF-7), for example, the localization of spectrin αII shows reduced cortical localization, resulting in decreased membrane stiffness compared to non-malignant MBE-F1 cells.^11^ In addition, the loss of spectrin may promote the epithelial–mesenchymal transition (EMT),^17,18^ a process critical for metastasis that involves profound changes in cell mechanics. These fragmented observations indicate that spectrin could be an important, but underappreciated, regulator of cancer cell mechanics.

The cytoskeleton regulates cell stiffness, which directly influences membrane topology. Reduced stiffness can induce structural changes, such as vesicle formation. Conversely, membrane topology can affect the cytoskeleton through proteins that sense membrane curvature. The Bin/Amphiphysin/Rvs (BAR)-domain superfamily, the most studied of the curvature-sensing proteins, shows widespread linkages between membrane morphology and the actin cytoskeleton.^19^ For instance, the F-BAR protein FBP17 regulates the actin filaments in conjunction with Arp2/3, and the N-BAR protein Bin1 directly stabilizes actin.^20,21^ Many studies have examined the interplay between actin and the cell membrane; however, the relationship between the spectrin network and membranes is much less well understood. Spectrin interacts with membranes both directly—primarily through the pleckstrin homology (PH) domain of its β-subunit—and indirectly *via* adaptor proteins such as ankyrin, thereby providing a platform that helps stabilize membrane architecture *in vivo*.^22,23^ However, to our best knowledge, no protein has been identified that links membrane curvature to the spectrin network in a manner analogous to BAR-domain proteins for actin. Interestingly, spectrin is absent from highly curved regions of the plasma membrane, such as lamellipodia and filopodia.^14^ This raises the possibility that spectrin-membrane interactions may differ in response to membrane morphology, or that an adaptor protein with a curvature-sensing property mediates such interactions.

Recently, proteomics-based approaches using artificially curved membranes, including our previously developed curvature-sensing assay, have enabled comprehensive identification of curvature-associated proteins.^24,25^ Using this methodology, spectrin αII and βII were identified as potential curvature sensors in MDA-MB-231 breast cancer cells and normal human dermal fibroblasts (NHDFs).^24^ However, this approach cannot clarify whether spectrin itself senses membrane curvature or if its localization is mediated by adaptor proteins with curvature-sensing capabilities. To address this question, direct *in vitro* reconstitution using purified spectrin and artificial membranes is required. Here, the contribution of the non-erythroid spectrin network to cancer cell stiffness is characterized using a microfluidic assay. Notably, this study highlights curvature-dependent changes in the spectrin-membrane interaction using both *in vivo* imaging analysis and an *in vitro* assay with artificial membranes of defined curvature. Such insights are important for understanding of the spectrin network's role in the regulation of cell membrane morphology and mechanical responses.

## Experimental

### Recombinant DNA

The pCIG-SPTAN1-FLAG plasmid was kindly provided by Dr Hirotomo Saitsu (Hamamatsu University School of Medicine). The plasmid encoding SPTBN1-HA was obtained from Addgene (#31070, MA, USA). For microscopic observation, the SPTBN1 gene was cloned into the pAcGFP-N1 vector (Takara Bio, Shiga, Japan) for fusion to the N-terminus of a green fluorescent protein (GFP) using SacII and HindIII restriction sites.

### Cell culture

HEK293T cells (ECACC 12022001) were obtained from the European Collection of Authenticated Cell Cultures (Salisbury, UK) *via* KAC Co., Ltd (Kyoto, Japan). MDA-MB-231 cells (ECACC 92020424) were purchased from Sigma-Aldrich (St. Louis, MO, USA).

For cell deformation cytometry and microscopic observation, MDA-MB-231 cells were cultured in Dulbecco's modified Eagle medium with sodium pyruvate (DMEM; Gibco, NY, USA; Cat# 11995-065) supplemented with 10% fetal bovine serum (FBS; Gibco), 100 U per mL penicillin–streptomycin (Fujifilm Wako Pure Chemical Corporation, Osaka, Japan). Cells were maintained at 37 °C in a humidified atmosphere containing 5% CO_2_ and passaged with 0.05% trypsin–ethylenediaminetetraacetic acid (trypsin–EDTA; Gibco) before reaching ∼80% confluency. When cells reached ∼70% confluency, they were transfected with 4 µg of plasmid DNA per 35-mm culture dish or 500 ng per 24-well plate using Lipofectamine 3000 (Thermo Fisher Scientific, MA, USA), following the manufacturer's instructions. The cells were analyzed 24–48 hours after transfection.

For the expression and purification of spectrin αII and spectrin βII, HEK293T cells were cultured as described above. Cells were transfected with 4 µg plasmid DNA per 35-mm culture dish using PEI-MAX reagent (Polysciences, PA, USA) and maintained for 48–72 h, with medium exchanged every 24 h.

### Protein purification and pull-down assay for the heterodimer

**Fig. 1:**
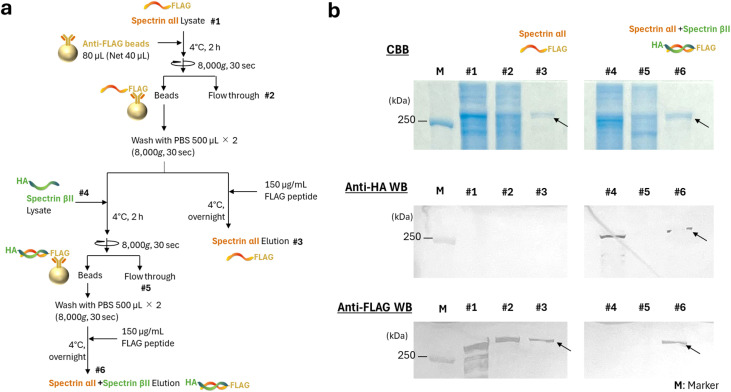
Purification and analysis of spectrin αII and βII. (a) Schematic diagram illustrating the affinity purification procedure for spectrin αII or βII proteins using affinity beads. (b) SDS-PAGE and western blotting analysis of the indicated fractions collected during the purification process.

FLAG-tagged spectrin αII proteins were expressed in HEK293T cells and collected from lysates using anti-DYKDDDDK tag antibody beads (Fujifilm Wako) (Fig. 1). Beads were incubated with the lysate for 2 h, washed three times with phosphate-buffered saline (PBS; Fujifilm Wako), and bound proteins were eluted with 150 µg mL^−1^ DYKDDDDK peptide (Fujifilm Wako) for 2 h. Excess peptide was removed using an 8 kDa Mini Dialysis Kit (Cytiva, Tokyo, Japan), and proteins were stored at 4 °C for up to 7 days.

To prepare spectrin αII/βII heterodimers, FLAG-tagged αII was immobilized on antibody beads and washed three times with PBS. Lysates from HEK293T cells expressing spectrin βII were applied to the bead suspension and incubated for 2 h to allow heterodimer formation. The beads were then washed three times with PBS, and heterodimers were eluted with peptide, followed by buffer exchange *via* dialysis. Purification was confirmed by 7.5% sodium dodecyl sulfate-polyacrylamide gel electrophoresis (SDS-PAGE) followed by Coomassie Brilliant Blue (CBB) R-250 staining (Bio-Rad, CA, USA). The presence of FLAG-tagged and HA-tagged proteins was further confirmed by western blotting (WB) using the iBlot2 dry blotting system (Thermo Fisher Scientific) according to the manufacturer's instructions. The DYKDDDDK tag monoclonal antibody (Proteintech Group, IL, USA) or the HA tag monoclonal antibody (Proteintech Group) diluted 1 : 2000 in iBind solution (Thermo Fisher Scientific) were used as the primary antibodies. The secondary antibody was Peroxidase AffiniPure goat anti-mouse IgG (H + L) (Jackson ImmunoResearch, PA, USA). After blotting, proteins were detected with Pierce 1-Step Ultra TMB Blotting Solution, Thermo Fisher Scientific) containing 3,3′,5,5′-tetramethylbenzidine (TMB).

### Cell deformability cytometry

Cell stiffness was measured using a previously reported microfluidic device-based method.^26^ Briefly, the microfluidic device was fabricated from polydimethylsiloxane (PDMS) using a silicon wafer mold with cross-flow channels created by lithography. The channel had a cross-section of 35 µm × 25 µm and was sealed at the bottom with a cover glass. MDA-MB-231 cells (1 × 10^6^ cells per mL), suspended in PBS containing 0.5% (w/v) methylcellulose (Sigma, Welwyn Garden City, UK), were introduced into the cross-flow channels from two opposing inlets at flow rates of 5–25 µL min^−1^. The device was mounted on an inverted bright-field microscope (Eclipse Ti–U; Nikon, Tokyo, Japan), and cell deformation at the junction was recorded using a high-speed camera (Photron, Tokyo, Japan) at 6–20 kfps. The cell deformation index (DI) was calculated by processing captured images of cells using MATLAB (MathWorks, MA, USA) and ImageJ software (NIH, MD, USA). DI was defined as *H*/*W*, where *H* and *W* represent the height and width of the cell at the point of maximum deformation within the cross-slot, respectively. DI values were plotted as a function of flow rate to assess cell stiffness. The DI was derived from high-speed video recordings and image analysis of 85 to 515 cells per experiment.

### Microscopic observation

MDA-MB-231 cells were transfected with a plasmid encoding SPTBN1-AcGFP using the lipofection method described above. Following transfection, cells were subjected to selection with 2000 µg per mL G-418 sulfate solution (Fujifilm Wako) for two weeks. Stable transfectants were seeded onto glass-bottom dishes and cultured for 24 h prior to observation. Cells were fixed with 4% paraformaldehyde (Fujifilm Wako) for 15 min at room temperature, permeabilized with 0.1% Triton X-100 (Sigma) for 3 min, and stained with Phalloidin-iFluor 647 (Abcam, Cambridge, UK) according to the manufacturer's instructions. Between each step, cells were washed three times with PBS. Fluorescence images were acquired using a Leica DMi8 microscope (Leica Microsystems, Wetzlar, Germany).

### Lipid strip assay for spectrin αII

A membrane lipid strip assay was performed to evaluate the interaction between spectrin αII and various lipids. The lipid strip (Echelon Biosciences, UT, USA) contained 15 different lipids and blank control spots. The strip was blocked with PBS containing 1% skim milk and 0.1% Tween-20 (PBS-T) for 1 h at room temperature. FLAG-tagged spectrin αII (1.0 µg mL^−1^) was applied and incubated for 1 h, followed by three washes with PBS-T. Bound proteins were detected by immunoblotting with *anti*-DYKDDDDK tag primary antibody followed by horseradish peroxidase (HRP)-conjugated anti-mouse secondary antibody (Proteintech Group, IL, USA), each incubated for 1 h. Chemiluminescence was developed using ImmunoStar LD reagent (Fujifilm Wako), and signals were imaged with an Amersham ImageQuant 800 ECL system (GE Healthcare, MA, USA). Spot intensities were quantified using ImageJ software.

### Fabrication of spherical supported lipid bilayers (SSLBs)

Artificial curved membranes were prepared as previously described,^24^ with minor modifications. A lipid mixture consisting of 0.5 mg of 1,2-dioleoyl-*sn*-glycero-3-phosphocholine (DOPC), 1,2-dioleoyl-*sn*-glycero-3-phosphoethanolamine (DOPE), cholesterol, and phosphatidylinositol-4,5-bisphosphate (PtdIns(4,5)P_2_) (Avanti Polar Lipids, AL, USA) at a molar ratio of 4 : 4 : 1 : 1 was hydrated in 0.5 mL of PBS and sonicated to generate unilamellar liposomes. Silica beads (2 mg, 1000 nm diameter; Micromod Partikeltechnologie, Rostock, Germany) were incubated with liposomes (80 µg) for 1 h at 45 °C to form supported spherical lipid bilayers (SSLBs). SSLBs of smaller diameters (100, 50, and 30 nm) were prepared in the same manner, maintaining a constant ratio of liposome mass to total bead surface area. Excess lipids were removed by centrifugation and resuspension in fresh PBS.

### Curvature-sensing evaluation using SSLB sedimentation assay

The sedimentation assay was performed on purified proteins using the previously described method,^27^ with SSLBs of 1000, 100, 50 and 30 nm in diameter. SSLBs (2 mg of 1000 nm) were mixed with either 5 µg of purified spectrin αII or 25 µg of αII/βII heterodimers and incubated overnight at 4 °C. To equalize the total surface area among different SSLB sizes, appropriate amounts of each SSLB preparation (100 nm: 200 µg; 50 nm: 100 µg; 30 nm: 60 µg) were added to the protein solutions. After incubation, samples were separated into bound and unbound fractions by centrifugation (20 000 g, 45 min). The supernatant was collected as the unbound fraction. The SSLB pellet was washed with PBS and resuspended in 30 µL Laemmli sample buffer (Bio-Rad, CA, USA) and heated at 95 °C for 5 min to dissociate membrane-bound proteins. After centrifugation, the released proteins in the supernatant were precipitated with 1 mL of cold acetone and designated as the bound fraction. Both fractions were analyzed by SDS-PAGE as described above. The proteins in each sample were measured using ImageJ software.

## Results and discussion

### Spectrin enhances cell stiffness in cancer cells

**Fig. 2:**
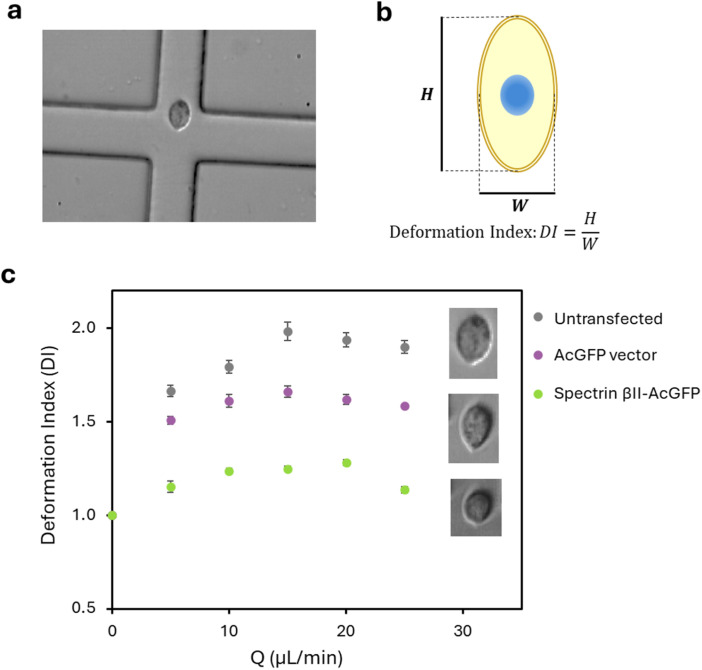
Cell deformability cytometry of MDA-MB-231 cells expressing spectrin βII. (a) Representative microscopic image of the cross-flow channel junction. (b) Deformation index (DI = H/W) extracted from high-speed videos of cell deformation. (c) Deformation index (DI) as a function of flow rate (Q) for non-transfected MDA-MB-231 cells (gray), cells transfected with the AcGFP vector (purple), and cells transfected with the Spectrin βII–AcGFP plasmid (green).

The mechanical properties conferred by spectrin βII were investigated using a microfluidic cross-flow deformability assay. To ensure that cell deformation did not cause irreversible damage, cell viability was evaluated after passage through the device. MDA-MB-231 cells were cultured for 24 hours after treatment, and their viability was measured against the control (Fig. S1). Cell viability exceeded 95% at flow rates up to 25 µL min^−1^, confirming that this assay can evaluate cell stiffness without irreversible damage. Based on this result, all subsequent experiments were conducted at a maximum flow rate of 25 µL min^−1^. To compare the stiffness of non-transfected and spectrin-expressing MDA-MB-231 cells, we assessed cellular stiffness by measuring deformation under compressive forces at the junction of the microfluidic device (Fig. 2). The deformation index (DI) was calculated from high-speed video recordings. Non-transfected cells exhibited higher deformation, reaching a DI value of approximately 2.0 at 15 µL min^−1^. In contrast, cells overexpressing spectrin βII exhibited a significantly lower DI of ∼1.2, indicating a more spherical and rigid shape. The empty vector did not show a significant increase in cell stiffness; DI values were higher than 1.5 even in the low flow rate region. Our results demonstrate that spectrin βII expression significantly increases cell stiffness. Collectively, these findings suggest that spectrin βII may be one of the key cytoskeletal proteins that modulate cell stiffness, a mechanical property that can influence the invasive potential of cancer cells.

### Spectrin networks stabilize on flat cell membranes

**Fig. 3:**
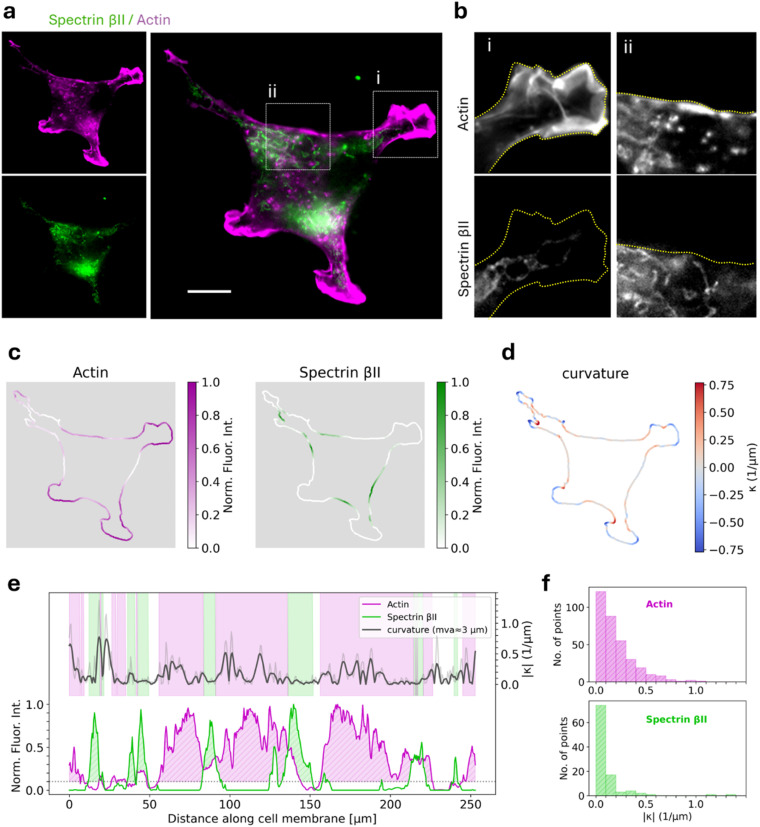
(a) Intracellular localization of GFP-tagged spectrin βII and actin in MDA-MB-231 cells. Scale bar: 10 µm. (b) Representative images of a highly curved membrane region (i) and a flat membrane region (ii). (c) Normalized fluorescence intensities of actin and spectrin βII along the cell boundary. (d) Membrane curvature (*κ*) of the cell outline. Positive curvature is defined as bending inward toward the cytoplasm, and negative curvature as the opposite. (e) Fluorescence intensity profiles of actin (purple) and spectrin βII (green) (left *y*-axis), and the absolute value of smoothed membrane curvature |*κ*| (gray, right *y*-axis), plotted counterclockwise from the top-left point of the cell. Purple and green shaded areas indicate actin- and spectrin-enriched regions, respectively. (f) Distribution of local membrane curvature values at sampling points located within actin-enriched and spectrin-enriched regions.

To investigate the interplay between the spectrin cytoskeleton, actin filaments, and plasma membrane morphology, the localization of spectrin and actin was observed in human breast cancer cells. MDA-MB-231 cells that stably express GFP-tagged spectrin βII were fixed and stained with phalloidin, and imaged using a fluorescence microscope (Fig. 3a). Note that, in this experiment the cells were adherent and exhibit membrane protrusions, giving irregular outlines. In most cells, spectrin βII was distributed throughout the cytoplasm in fibrous or patchy structures. Spectrin βII was clearly excluded from highly curved membrane regions, such as the actin-rich leading edge (Fig. 3b), consistent with a previous report in mouse embryonic fibroblast cells.^14^ To quantitatively examine the relationship between local plasma membrane curvature and the localization of spectrin or actin, we performed imaging analysis to extract the cell periphery (Fig. 3c). Cells displayed complex membrane structures with high curvature at protrusions (Fig. 3d). While these regions are dominated by actin, spectrin localized preferentially on flatter membranes with lower curvature (Fig. 3e and f). These observations suggest that the spectrin-membrane interaction is likely modulated by local membrane topology.

### Preferential interaction of spectrin heterodimers with flat membranes

**Fig. 4:**
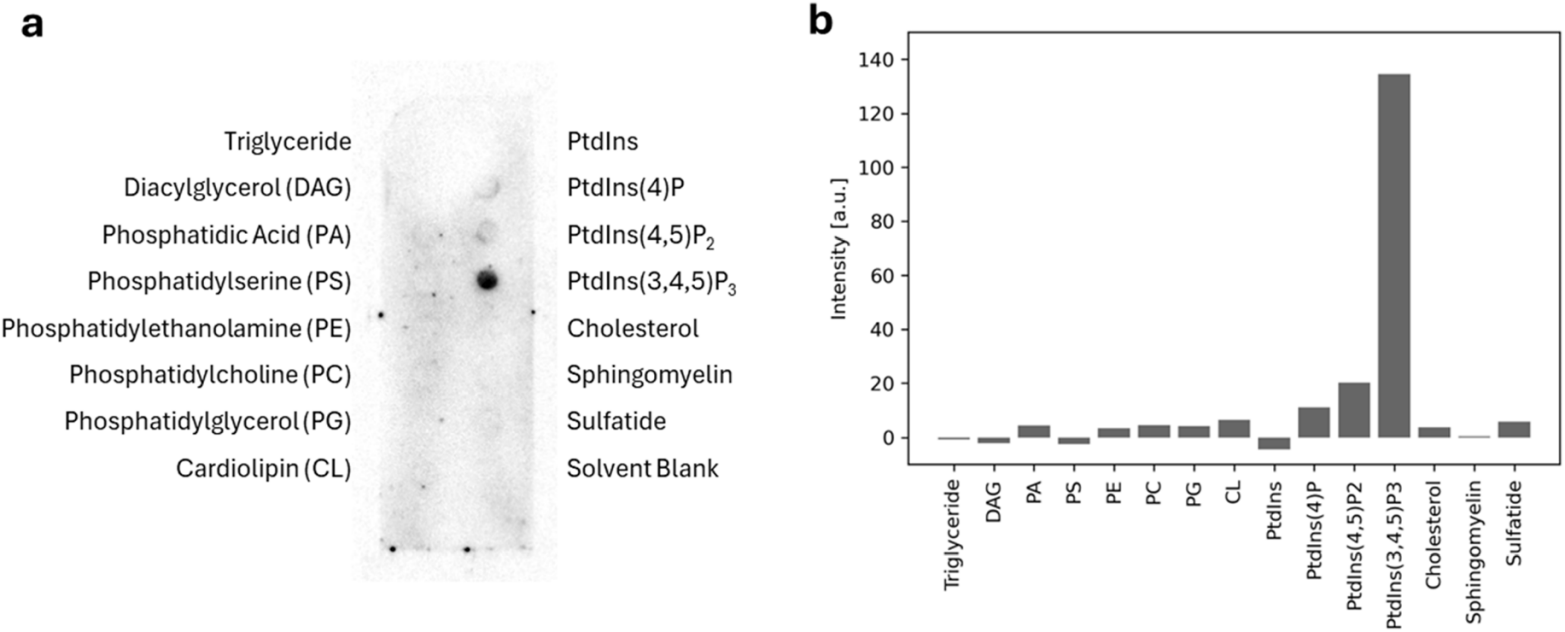
A lipid strip assay of FLAG-tagged spectrin αII. (a) Chemiluminescence image of the membrane lipid strip with 15 immobilized lipids detected by immunoassay. (b) Relative spot intensities quantified by image analysis.

To determine whether the localization of spectrin to flat cell membranes, as observed in the previous section, is due to direct spectrin-membrane interaction or adapter protein mediation, an *in vitro* assay with purified proteins was conducted. Of the two subunits that compose the heterodimer, spectrin βII binds to the cell membrane *via* its N-terminal PH domain, which exhibits promiscuous affinity for phosphoinositides.^28^ A previous study confirmed the binding activity of spectrin βII to several phosphoinositides, specifically phosphatidylinositol 4,5-bisphosphate (PtdIns(4,5)P_2_), phosphatidylinositol 3,4-bisphosphate (PtdIns(3,4)P_2_), and phosphatidylinositol 3,4,5-trisphosphate (PtdIns(3,4,5)P_3_), by means of a lipid strip assay. Critically, the K2207Q mutation in the PH domain of spectrin βII was found to abolish this phosphoinositide binding.^29^ However, studies on the direct membrane interaction of the non-erythrocyte spectrin αII are limited. To investigate its lipid-binding properties, we performed a lipid strip assay using purified, FLAG-tagged spectrin αII. The protein was incubated with a membrane lipid strip spotted with 15 distinct lipids. Bound spectrin αII was then detected by immunostaining with an anti-FLAG antibody. As shown in Fig. 4, spectrin αII exhibited a relatively high affinity for phosphoinositides, with particularly strong binding to PtdIns(3,4,5)P_3_. This result suggests that both the PH domain of the βII subunit and the αII subunit interact with the membrane *via* phosphoinositides.

**Fig. 5:**
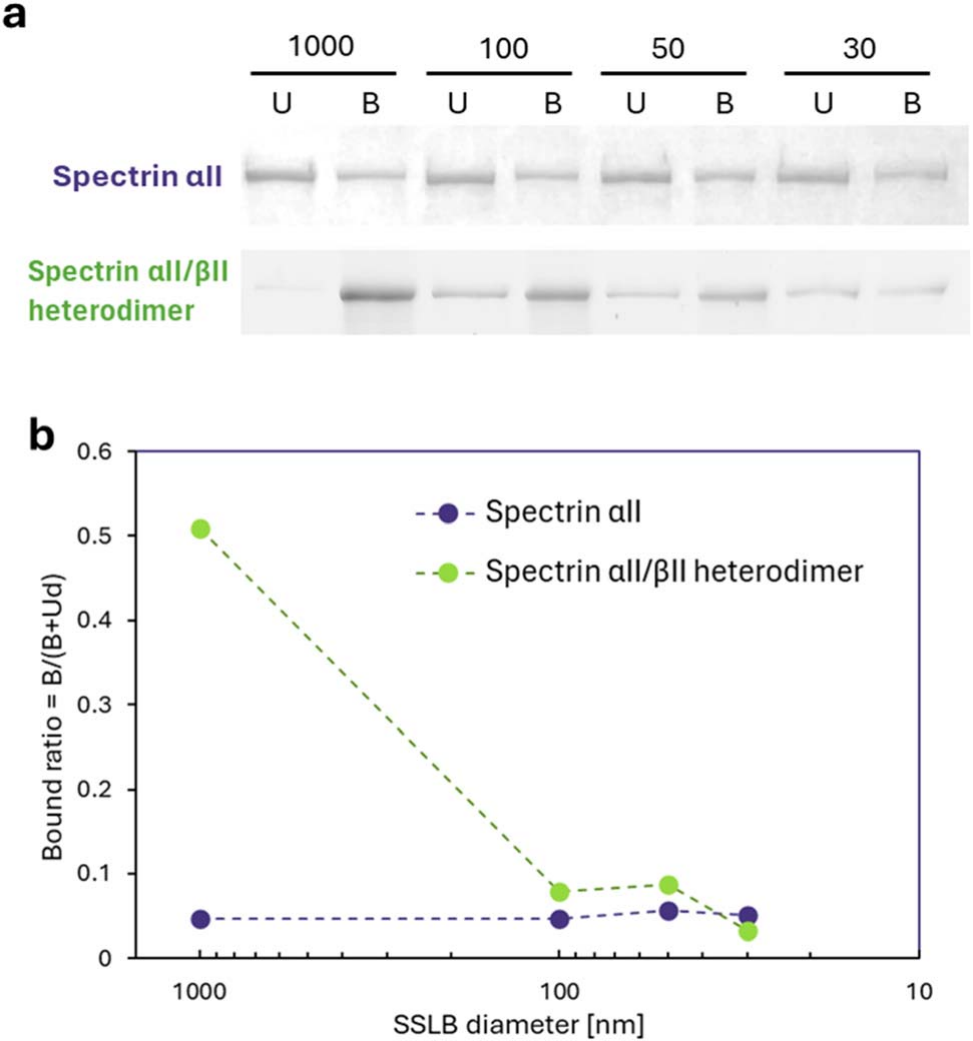
Sedimentation assay of SSLBs with purified spectrin αII or αII/βII heterodimer. (a) SDS–PAGE of bound (B) and unbound (U) fractions. (b) Relative binding ratios on SSLBs of different diameters (B, intensity of bound fraction; U, intensity of unbound fraction; d, dilution factor of U).

To evaluate the influence of membrane morphology on these interactions, we incubated either purified spectrin αII or the αII/βII heterodimer with supported spherical lipid bilayers (SSLBs) of various sizes, then compared the amount of bound protein. First, the heterodimers were reconstituted on affinity beads from HEK293T cell lysates in which the αII and βII subunits were overexpressed separately. The detection of the FLAG tag derived from spectrin αII and the HA tag derived from spectrin βII by western blotting confirmed the reconstitution of the heterodimer on affinity beads (Fig. 1). Subsequently, the proteins were incubated with SSLBs of various diameters (30, 50, 100, and 1000 nm) that were prepared to have identical total surface areas. The amounts of bound and unbound protein were then quantified using SDS-PAGE. The ratio of bound spectrin αII was nearly constant across all SSLB sizes, suggesting that its interaction with the membrane is independent of curvature (Fig. 5). In contrast, the αII/βII heterodimer exhibited a markedly higher affinity for flat membranes. The proportion of protein bound to 1000-nm SSLBs was approximately 15 times greater than the proportion bound to the highly curved 30-nm SSLBs. This result indicates that the heterodimeric state confers a potent membrane curvature-sensing ability, which is likely the primary reason for the intracellular localization of spectrin to flat membrane regions.

Our findings reveal a direct link between membrane topology and spectrin-membrane interactions. Specifically, spectrin networks were more stably associated with flat membranes than with highly curved regions, both *in vivo* and *in vitro* assays. Indeed, the curvature of 1000-nm SSLBs (|*κ*| ≈ 0.5 µm^−1^) is broadly consistent with the preferred curvature for spectrin localization observed *in vivo* (|*κ*| < 0.2 µm^−1^) (Fig. 3f). Previous *in vitro* studies using giant unilamellar vesicles have suggested that the spectrin–actin network forms a scaffold that mechanically reinforces the lipid bilayer, thereby affecting membrane morphology.^30,31^ In contrast, our findings offer a complementary view: membrane geometry modulates spectrin–membrane interactions, thereby determining its spatial localization. This interaction appears to depend on the oligomerization of spectrin heterodimers. Additionally, our results showed that both the αII and βII subunits interact with phosphoinositides, including PtdIns(4,5)P_2_ and PtdIns(3,4,5)P_3_. While BAR-domain proteins also bind to these lipids,^32^ spectrin differs in its preference for low-curvature, flat membranes over highly curved ones. This contrast suggests that spectrin and BAR-domain proteins may act in a complementary manner: BAR-domain proteins initiate curvature formation at the nanometer scale, whereas spectrin stabilizes planar membrane domains at the micron scale. These specialized roles could underlie dynamic remodeling of the plasma membrane between protrusive and stable membrane regions during cell morphogenesis.

One possible explanation is that spectrin requires oligomerization to sense curvature at the micron scale, a mechanism conceptually similar to septins, which associate with membranes only after polymerization.^33^ Such topology-dependent remodeling may help maintain cellular stiffness. Beyond curvature-sensing, this property positions spectrin as a potential integrator of mechanical tension and membrane topology—acting as a mechanosensor that stabilizes the cortex under tensile stress by preventing excessive curvature formation. In this framework, spectrin networks could serve as mechanical modulators, coupling local membrane flatness to overall cortical homeostasis.

The stabilization of the spectrin network on flat membranes is likely an important determinant of cellular stiffness. In cancer, spectrin βII is often downregulated,^16^ which may disrupt this network and contribute to the reduced stiffness and enhanced invasiveness observed in metastatic cells,^5^ or subsequently facilitate EMT. During EMT, cells undergo dramatic cytoskeletal remodeling and adopt a more deformable phenotype; the loss of spectrin-mediated membrane stabilization could consequently promote this transition by allowing enhanced cortical fluidity and bleb-based migration. In this sense, spectrin downregulation may not simply correlate with malignancy but could play a major role in the acquisition of mechanical plasticity characteristic of invasive cancer cells. Consistent with this idea, our data show that spectrin βII overexpression increases the stiffness of MDA-MB-231 cells. Furthermore, a previous proteomic work has demonstrated that spectrin from cancer cells binds preferentially to highly curved membranes (50–100 nm), while spectrin from normal cells binds to flat membranes.^24^ This difference suggests that malignant transformation alters spectrin's intrinsic curvature preference, potentially through changes in oligomerization or post-translational modifications. Such a shift may perturb the functional balance between spectrin and BAR-domain proteins, which normally cooperate at different spatial scales to maintain membrane curvature homeostasis. Disruption of this balance might promote aberrant remodeling of the plasma membrane, potentially predisposing cells to increased deformability and reduced cortical reinforcement. These alterations could gradually reprogram the mechanical landscape of cancer cells, weakening cortical stability and enhancing morphological adaptability during invasion and metastasis. Collectively, these results highlight spectrin as a potential mechanosensor that regulates cell stiffness and whose altered behavior may underlie the pathological mechanics of cancer cells.

## Conclusion

In summary, this study identifies three key mechanisms underlying spectrin function. First, our *in vitro* reconstitution assays demonstrate that spectrin senses membrane curvature, preferentially associating with flat membranes—a property that strictly depends on αII/βII heterodimer formation. Second, we show that, in addition to βII, the αII subunit specifically interacts with phosphoinositides (PtdIns(3,4)P_2_ and PtdIns(3,4,5)P_3_). Consistent with these *in vitro* findings, curvature-dependent spectrin localization was also observed *in vivo*. Functionally, cell deformation cytometry revealed that spectrin expression significantly increases the overall stiffness of breast cancer cells. Together, these results support a model in which spectrin acts as a curvature-sensitive cortical scaffold that stabilizes flat membrane domains. Loss of this stabilization may contribute to the reduced stiffness and increased deformability characteristic of EMT, suggesting that spectrin plays a critical mechanosensory role in cancer cells. Ultimately, targeting spectrin-mediated membrane stabilization may offer a potential future therapeutic strategy to prevent mechanical softening and inhibit cancer metastasis.

## Author contributions

Takumi Komikawa: conceptualization, methodology, investigation, writing – original draft. Takahiro Miyake: conceptualization, methodology, investigation. Mayu Morooka: conceptualization, methodology, investigation. Rikuto Kawakami: methodology, investigation. Shogo Saito: methodology, resources, writing – review & editing. Kevin Critchley: supervision, funding acquisition, writing – review & editing. Stephen D. Evans: supervision, funding acquisition, writing – review & editing. Mina Okochi: conceptualization, supervision, funding acquisition, writing – review & editing. Masayoshi Tanaka: conceptualization, supervision, funding acquisition, writing – review & editing.

## Conflicts of interest

The authors declare no conflict of interest.

## Supplementary Material

RA-015-D5RA08200E-s001

## Data Availability

The data supporting this article have been included as part of the supplementary information (SI). The Python scripts used for image analysis are available at GitHub (https://github.com/takumi485/spectrin-curvature-analysis). Supplementary information is available. See DOI: https://doi.org/10.1039/d5ra08200e.
