# Supplementary material for: Non-erythrocyte spectrin network preferentially stabilizes flat membrane and enhances cell stiffness
Source: RSC Adv. 2025 Dec 12;15(58):49661–8. doi: 10.1039/d5ra08200e (PMC12699370; doi:10.1039/d5ra08200e)
Supplement: RA-015-D5RA08200E-s001 [file RA-015-D5RA08200E-s001.pdf]

Supplementary Information (SI) for RSC Advances.
This journal is © The Royal Society of Chemistry 2025

Electronic Supporting Information for *RSC Advances*

Non-erythrocyte spectrin network preferentially stabilizes flat membrane and enhances cell stiffness

Takumi Komikawa[1], Takahiro Miyake[2], Mayu Morooka[2], Rikuto Kawakami[1], Shogo Saito[2], Kevin Critchley[3], Stephen D. Evans[3], Mina Okochi[2,*], Masayoshi Tanaka[1,*]

[1]School of Materials and Chemical Technology, Institute of Science, Tokyo, 4259 Nagatsuda, Midori-ku, Yokohama-city, Kanagawa 226-8503, Japan
[2]School of Materials and Chemical Technology, Institute of Science Tokyo, 2-12-1 O-okayama, Meguro-ku, Tokyo 152-8552, Japan
[3]School of Physics and Astronomy, University of Leeds, Leeds LS2 9JT, United Kingdom

***Corresponding Authors:**
Masayoshi Tanaka
Email: tanaka.m@mct.isct.ac.jp
Tel/FAX: +81-45-924-5567
Mina Okochi
Email: okochi.m.aa@m.titech.ac.jp
Tel/FAX: +81-3-5734-2116

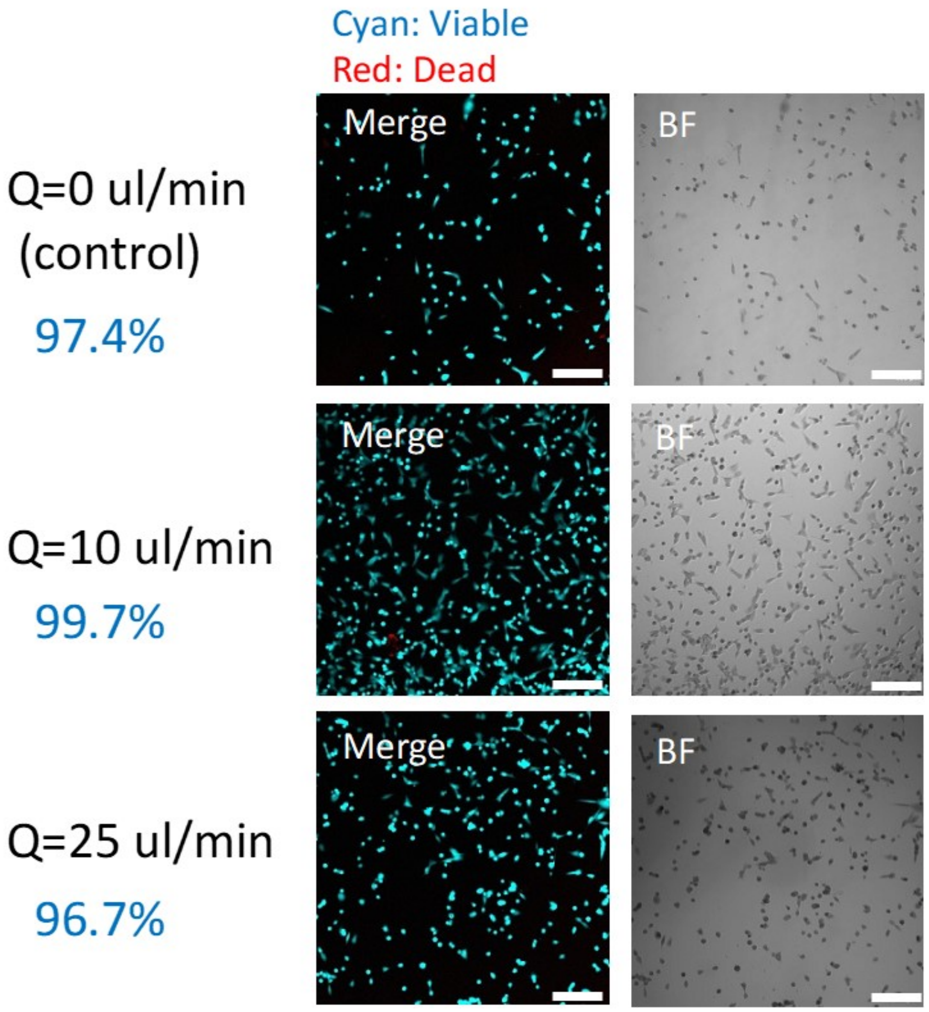

Fig. S1 Cell viability of MDA-MB-231 cells after passage through the microfluidic cross-flow device. MDA-MB-231 cells were cultured for 24 hours post-treatment with varying flow rates (Q) and stained with Calcein-AM (live cells, cyan) and NucRed647 (dead cells, red). Cell viability was calculated from image analysis. Scale bar: 200 μm.

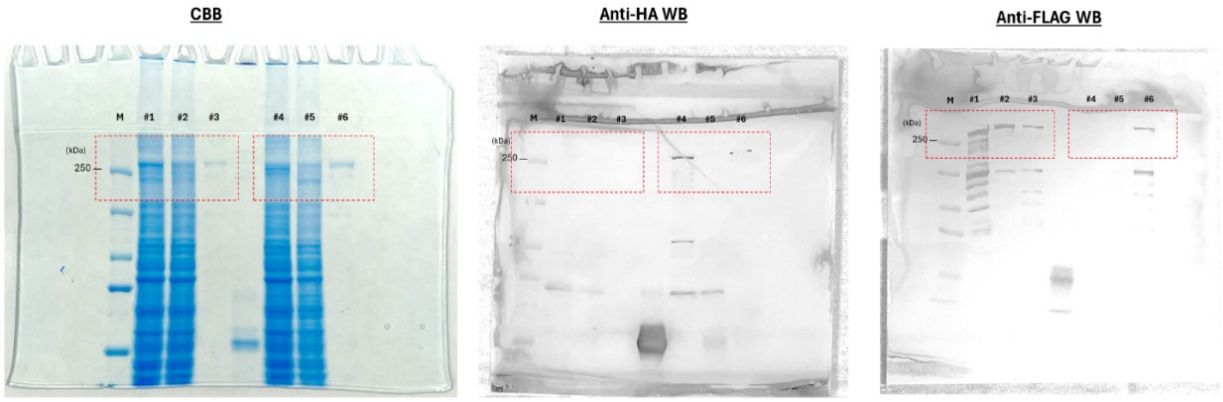

Fig. S2 Full-length gel or membrane images corresponding to Fig. 1b in the main text. The red boxes indicate the regions used in the main figure.

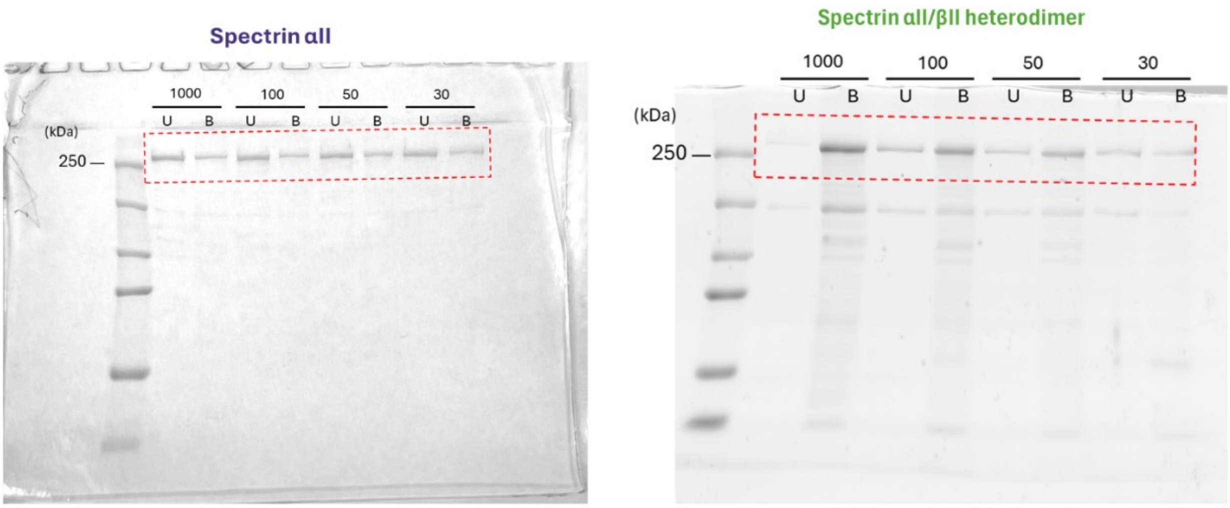

Fig. S3 Full-length gel images corresponding to Fig. 5a in the main text. The red boxes indicate the regions used in the main figure.
